# The SSX Family of Cancer-Testis Antigens as Target Proteins for Tumor Therapy

**DOI:** 10.1155/2010/150591

**Published:** 2010-10-11

**Authors:** Heath A. Smith, Douglas G. McNeel

**Affiliations:** ^1^Departments of Medicine and Oncology, University of Wisconsin-Madison, Madison, WI 53705, USA; ^2^7007 Wisconsin Institutes for Medical Research, 1111 Highland Avenue, Madison, WI 53705, USA

## Abstract

Cancer-testis antigens (CTAs) represent an expanding class of tumor-associated proteins defined on the basis of their tissue-restricted expression to testis or ovary germline cells and frequent ectopic expression in tumor tissue. The expression of CTA in MHC class I-deficient germline cells makes these proteins particularly attractive as immunotherapeutic targets because they serve as essentially tumor-specific antigens for MHC class I-restricted CD8+ T cells. Moreover, because CTAs are expressed in many types of cancer, any therapeutic developed to target these antigens might have efficacy for multiple cancer types. Of particular interest among CTAs is the synovial sarcoma X chromosome breakpoint (SSX) family of proteins, which includes ten highly homologous family members. Expression of SSX proteins in tumor tissues has been associated with advanced stages of disease and worse patient prognosis. Additionally, both humoral and cell-mediated immune responses to SSX proteins have been demonstrated in patients with tumors of varying histological origin, which indicates that natural immune responses can be spontaneously generated to these antigens in cancer patients. The current review will describe the history and identification of this family of proteins, as well as what is known of their function, expression in normal and malignant tissues, and immunogenicity.

## 1. Introduction

Theclass of proteins known as cancer-testis antigens (CTAs) are a subgroup of tumor proteins with normal expression found almost exclusively in testis germline tissues and aberrant expression in many types of cancer [1]. More than 110 CTA genes have been identified to date, with approximately 30 members encoded by genes located on the X chromosome, frequently in multigene families [2]. These CTA genes located on the X chromosome, called *CT-X* genes, are predicted to comprise ~10% of the genes encoded by X chromosomal DNA, and many of these *CT-X *genes have homologues in mice that are also located on the X chromosome and restricted in expression to testis tissues [2–4]. Typically, the *CT-X* genes cluster in two chromosomal regions, at a telomeric region between Xq24 to Xq28 and a centromeric region from Xp11.2 to 11.4. 

In addition to their tissue-restricted expression patterns, CTA proteins share a number of other common characteristics. For instance, many are encoded by multigene families, can be epigenetically regulated in expression level with drugs such as 5-aza-2′-deoxycytidine, and many have unknown functionality yet appear to play some role in tumorigenesis. Additionally, most CTAs have heterogeneous expression in cancer tissues and are frequently expressed in high-grade or late tumor stages, with expression often correlated with a worse prognosis. Tumors expressing one CTA are also often found to express multiple CTAs, and several have been found to be targets of spontaneous humoral or cell-mediated immune responses [2]. The majority of CT-X antigens are expressed in the testis at the spermatogonia stage of spermatogenesis [5], whereas other CTAs appear to be restricted in expression to haploid cells [3]. Most non*CT-X *genes are single-copy genes with no chromosomal clustering, and expression patterns often not entirely restricted to MHC-deficient germ cells or cancer tissue. 

While these proteins represent potential therapeutic targets for cancer based upon their predominant expression selectively in tumor tissues, they are particularly attractive as targets for tumor immunotherapy, specifically due to their preferential expression in immune-privileged testis tissue. Several physical and molecular mechanisms contribute to the immunoprivileged nature of testis tissue including localized cytokine-mediated immune suppression, antigen-specific immunoregulation, the presence of the blood-testis barrier, and an absence of MHC class I molecules on testis germ cells [6, 7]. The lack of MHC class I on the surface of germline cells means these cells are unable to present endogenous peptides to the host CD8+ T cells, suggesting that testis-specific proteins should be recognized as neo-antigens when expressed ectopically in tumor tissue elsewhere in the body. While the immune-privileged nature of testis tissue will likely result in decreased peripheral immune tolerance to CTAs expressed in tumors, it should be noted that some central tolerance to these antigens may be present since CTA proteins could be expressed in lymphoid tissues such as medullary cells of the thymus during T-cell selection [8]. However, CTAs encoded by the *CT-X* genes appear to be the most promising CTAs as therapeutic vaccine targets since their expression is most highly restricted to only testis and cancer tissues compared to other nonCT-X CTAs. 

 To date, only a few CTAs have been shown to elicit both humoral and cell-mediated immune responses in humans, including SSX, MAGE-A1, MAGE-A3, and NY-ESO-1 [2]. These proteins are currently the most promising CTA targets for tumor immunotherapy, and several clinical trials are underway to evaluate the efficacy of MAGE-A3 and NY-ESO-1 as tumor targets [9, 10]. Alternatively, many other proteins have been evaluated as potential targets for tumor immunotherapy including tissue differentiation antigens such as tyrosinase, Melan A/Mart-1, gp 100, and prostatic acid phosphatase (PAP) or antigens expressed at low levels in normal tissues and high levels in tumor tissue such as HER2 and Muc 1, [11–13]. These proteins may represent attractive targets for certain specific types of cancer, however, the SSX proteins are targets potentially applicable to many types of cancer instead of tumors of a restricted histological type. Additionally, the need to overcome preexisting immune tolerance to non CTA may represent a larger immunotherapeutic obstacle for antigens not expressed in immune-privileged tissue. While SSX proteins appear to be very promising targets for tumor therapy, the work evaluating this family as targets for cancer is less advanced compared to the MAGE or NY-ESO-1 proteins. 

 This paper will focus on the SSX family of cancer testis antigens. Recently these proteins have been identified as high priority targets for cancer therapy based upon certain predefined criteria such as antigen specificity, oncogenicity, expression level, and number of identified epitopes [14]. Some members of this family can become fused to the SS18 protein in synovial sarcoma (SS) through gene translocations, which is how these proteins were first identified. Interestingly, the SS18-SSX fusion genes are expressed in >95% of SS and appear to directly contribute to the cancer phenotype [15]. Much work has been conducted in the last decade to characterize this family of proteins, and although a great deal of information has been elucidated regarding their expression patterns, functionality, cellular localization, and immunogenicity, their role in cancer is still incompletely understood. The information obtained from these investigations has only emphasized the importance of SSX proteins as tumor targets.

## 2. SSX Identification

The SSX gene family was first identified through cytogenetic studies of synovial sarcoma (SS) in which approximately 70% of both biphasic and monophasic SS tumor types were found to contain the same characteristic chromosomal translocation event *t*(X;18)(p11.2;q11.2) [16–19]. By screening a cDNA library derived from a SS cell line with a yeast artificial chromosome (YAC) probe spanning the *t*(X;18) chromosome breakpoint, Clark et al. identified two novel genomic fragments by Southern blot analysis. Both of these transcripts, when sequenced, failed to exhibit strong homology to any published genomic sequences and were thus designated SYT for “Synovial Sarcoma Translocation” (now known as SS18 for Synovial Sarcoma gene of chromosome 18) and SSX for “Synovial Sarcoma X chromosome breakpoint” [20]. It was further found that the C-terminus of SSX was fused with the N-terminus of SS18, which indicated that the translocation would result in an SS18-SSX fusion protein. Because of the presence of this fusion event in such a high percentage of SS, it was predicted that this fusion protein must have inherent transforming activity independent of the normal function of the SS18 and SSX proteins.

Sequencing additional SS cDNA clones of this fusion site, Crew et al. found that the C-terminal regions of two distinct genes, designated SSX1 and SSX2, can become fused to the N-terminus of SS18 [21]. In the case of both genes, it was observed that the last 78 C-terminal amino acids of SSX1 and SSX2 replaced the last 8 amino acids of the C-terminus of SS18 in most tumors, however, alternate fusions were also observed less frequently for some tumor specimens. Based upon their predicted open-reading frames, these two genes were expected to encode proteins of 188 amino acids, exhibit ~81% protein sequence identity, contain consensus sequences for both N-glycosylation and tyrosine phosphorylation, be rich in charged amino acids (40%-41%), and in particular both proteins were found to have acidic C-terminal tails that are sometimes hallmarks of nuclear proteins. Using a nonspecific SSX probe, they also found by Northern blot that SSX1 and SSX2 expression was restricted to thyroid and testis tissues. 

 Türeci et al. later identified SSX2 as the cancer testis antigen (CTA) HOM-Mel-40 when it was found that some melanoma patients have IgG antibody immune responses specific for SSX2 using the serological analysis of recombinant cDNA expression libraries (SEREX) methodology [22]. By screening a cDNA library derived from melanoma tissue with sera from multiple melanoma patients it was found that high-titer IgG antibodies to HOM-Mel-40/SSX2 were found in approximately 10% of patients with melanoma (10/89), whereas SSX2 responses were absent in sera from healthy controls (0/41). In the same report, it was found that out of 32 tissues evaluated for SSX2 expression by RT-PCR and Northern Blot, SSX2 expression was restricted to testis tissue, and contrary to the report by Crew et al. expression in the thyroid was barely detectable by RT-PCR, whereas SSX2 expression was found in the testis by both Northern blot and RT-PCR. These results confirmed that SSX2 is essentially testis-specific among normal body tissues. Türeci also reported in the same paper that SSX2 was expressed in about 50% of melanoma tumor samples, 30% of hepatocellular carcinoma tumor samples, 25% of colon and prostate cancer samples, 20% of breast cancers, and several other tumor samples of different histological origin examined by RT-PCR. These findings firmly established the inclusion of SSX2 as a member of the CTA class of proteins. The presence of SSX2 expression in so many tumor types suggested that this antigen is upregulated in cancer independently from fusion events with SS18, perhaps by alterations in methylation status or by the overexpression of SSX2 transcriptional activators. In fact, it has been shown cytogenetically by several groups that the fusion member derived from the X chromosome (“der(X)” or SSX2) rather than the derivative member from chromosome 18 (“der(18)” or SS18) is important for maintaining proliferation and that der18 can be lost during the course of SS progression suggesting that the expression of the C-terminal portion of SSX may be the dominant event leading to tumor progression [16, 17, 19, 23, 24]. 

## 3. A Family of Proteins

A short time after the identification of the two SSX proteins involved in the SSX-SS18 fusion event it became clear that SSX1 and SSX2 were part of a larger family of homologous proteins, numbered sequentially based on their discovery. A study by Chand et al. using a pulse-field analysis of digested YACs spanning the X chromosome (OATL1 loci), revealed that perhaps as many as five copies of the SSX gene were present in this region. The analysis indicated that SSX or this genetic locus had undergone a series of duplication events [25]. Following on these observations, de Leeuw and colleagues identified a third SSX family member designated SSX3 by screening a testis cDNA library [26]. This gene had 90% identity to SSX1 and 95% identity to SSX2 at the nucleic acid level. Additionally, somatic cell mapping studies indicated that this third family member was located in the same region of the X chromosome (Xp11.2 → p11.1) as SSX1/2. However, in contrast to SSX1/2, RT-PCR data from 44 SS tumor samples revealed that SSX3 was not a fusion partner with SS18. 

SSX family members SSX4 and SSX5 were subsequently identified by Southern blot analysis using PCR and restriction map analysis [27]. Human genomic DNA was probed using a ^32^P-labeled SSX cDNA and the genomic DNA pulled out was sequenced for identification. The two new members brought the total membership of the SSX protein family to five, including a shorter SSX4 transcript representing an alternative splice. These family members shared 88–95% nucleotide homology and 77–91% amino acid homology. Cloning and sequencing of SSX2 as a prototypic SSX gene revealed a coding region containing six exons spanning a total genomic distance of 8 kb. RT-PCR showed that all five members were expressed in the testis. Further PCR analysis of melanoma cell lines indicated common expression of SSX1 and SSX2 (3/12 lines), while SSX4 and SSX5 detection was more rare (1/12), and SSX3 expression was not detected [27]. 

Not long after the first five homologues were described, a sixth family member, SSX6, was identified through database searches by dos Santos et al. [28], and three additional unique members were identified by Güre et al. through the screening and sequencing of a placenta genomic library with an SSX probe and extensive database analysis [29]. This brought the SSX gene family to a total of 9 members. In these database queries, ten additional SSX pseudogenes were identified, all of which mapped to chromosome X with the exception of *ψ*SSX10 found on chromosome 6. The protein homology of these family members ranges from 73%–92%, and the cDNA homology was found to be between 87%–96%. 

Each SSX homologue was found to encode the KRAB-A domain on two exons (4 and 5) and the SSX repression domain (encoded by exon 9), described below, and each complete gene occupied 8–10 kb with the distance between genes on the X chromosome ranging from 10–50 kb. However, in contrast to their previous study, which found that SSX members have 6 coding exons, further analysis of expressed sequence tags (ESTs) in GenBank revealed additional 5′ and 3′ exons in untranslated regions. This finding brought the total number of SSX exons to 10; however, it was found that only 8 of the 10 exons are utilized by all SSX members. From these sequencing studies, the authors confirmed that all members share conserved exon/intron junctions, exon sizes, and most introns, with a few exceptions for alternative splice isoforms (i.e., SSX2, SSX4, SSX5 and SSX7). SSX1–9 were all predicted to encode proteins of 188 amino acids except for SSX8 which has a premature termination in the 7th exon and is predicted to encode a shorter protein of 142 amino acids. Protein expression of SSX8 as well as the alternative splice variants for SSX2, 4, 5, and 7 have not been shown. With the exception of *ψ*SSX10 on chromosome 6, all SSX genes and pseudogenes were found to cluster within two loci on the X chromosome 2 mb apart. Based on the observed clustering of SSX family members within the contigs available in GenBank, Güre and colleagues suggested that the two main SSX gene clusters arose from a large duplication spanning an approximately 100 kb region [29]. A tenth SSX family member, designated SSX10 and predicted to encode a protein product, has been annotated in GenBank (GeneID: 100128582), but to date no information about its expression in normal or malignant tissues has been described. 

**Figure 1:**
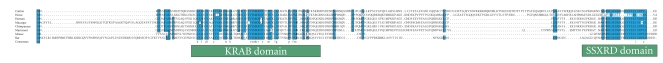
Protein homology and conserved domains among SSX orthologues. Shown is a sequence alignment of species-specific SSX protein sequences from canine, horse, human, macaque, chimpanzee, marmoset, mouse, and rat sources. Blue highlighting represents >75% sequence identity with consensus sequence shown on the bottom. The KRAB and SSXRD domain regions denoted by solid green bars are shown below the sequence alignment.

 The discovery that the SSX family contains several protein members is very characteristic of other CT-X antigens described thus far. For instance, it has been shown that the MAGE-A family consists of 12 members, GAGE 16 members, NY-ESO 3 members, and the SPANX family has 12 members, just to name a few of the more than 110 CTAs identified to date [2]. Not only have many SSX homologues been defined in humans, but several orthologues have been annotated for other species as well. The known SSX orthologues appear to be restricted to mammalian genomes with several SSX orthologues filed in the NCBI GenBank database including *Homo sapian *(human; accession XP_001128182), *Pan troglodytes* (chimpanzee; accession XP_001137291), *Macaca mulatta* (Macaque; accession XP_001103483), *Callithrix jacchus* (Marmoset; accession XP_002762880), *Canis lupus familiaris* (domesticated dog; accession XP_855346), *Equus caballus* (horse; accession XP_001917891), *Rattus norvegicus* (Norway rat; accession XP_002727549), and *Mus musculus* (common house mouse; accession EDL33981.1). All of these orthologues share the SSX repression domain (SSXRD), described below, and KRAB domains that are common among human SSX (hSSX) family members, with the SSXRD being the most highly conserved (Figure 1). 

 Several of the identified SSX orthologues, like human SSX (hSSX), appear to have homologous members within a species. For instance, a family of as many as 13 murine homologues have been well characterized on the mouse X chromosome [30]. These genes were first identified by Chen et al. by sequencing cDNA clones from mouse testis and tumor tissues. The mouse homologues were divided into two distinct classes, “Ssxa” and “Ssxb,” based on their sequence homologies. Ssxa consists of one member (Ssxa1), while Ssxb was found to have 12 members (Ssxb1–12). Ssxb family members were highly homologous overall with 75–98% nucleotide homology and 67–99% protein homology, however Ssxa and Ssxb were only approximately 30% homologous to each other, showing relatively large diversity between these two classes. These orthologues were located on chromosome X:cM position 5.9, and interestingly, this region is syntenic with human chromosome Xp11.3–p11.23 where the hSSX family members cluster. Again, like hSSX, the conserved SSXRD and KRAB domains are encoded by one and two exons, respectively. All intron sizes are similar between human and mice SSX genes, and all SSX genes span genomic regions of 8 kb. Also, like hSSX family members, some mouse (mSSX) homologues were expressed in murine tumors (predominantly Ssxb1 and Ssxb2), whereas others were not, and Ssxa and Ssxb mRNA expression was restricted to testis tissue among other normal tissues. Overall, mSSX genes are less than 35% homologous to hSSX, however, the KRAB and SSXRD domains were found to be as much as 49–66% homologous depending on the hSSX/mSSX members being compared [30]. The distinct similarities in expression patterns and other characteristics shared between mSSX and hSSX suggest that these orthologues may serve as useful models systems for preclinical tumor therapy studies.

## 4. SSX Function

### 4.1. Domain Functions and Cellular Localization

Like most CTAs, the function of SSX proteins, as well as the fusion partner SS18, is still not fully understood. It was determined from the initial identification of SSX that these proteins have KRAB-like domains and consensual sequences for N-glycosylation and tyrosine phosphorylation [21, 26]. Since SSX proteins also have an acidic tail [21] and a KRAB domain shown to have transcriptional repressor activity in a subgroup of zinc finger proteins, it was surmised that SSX may be involved in transcriptional regulation [31, 32]. Some of the first work characterizing the function of SSX proteins was carried out by dos Santos et al. evaluating the subcellular localization of FLAG- or VSV-tagged SS18, SSX, and the SS18-SSX2 fusion constructs using specifically-designed polyclonal antibodies [33]. They found that all three proteins were expressed in the nucleus of COS-1 cells transfected with constructs encoding cDNAs for these proteins. SSX2 had a diffuse pattern of expression in the nucleus, while SS18 and SS18-SSX2 had more punctate nuclear expression patterns [33], with no expression found in nucleoli. Staining of SS cells or HeLa cells with these antibodies revealed a punctate pattern of expression similar to that found for SS18- and SS18-SSX-transfected COS-1 cells. Double labeling of SS18 or SS18-SSX proteins with other known nuclear domains in COS-1 cells revealed no significant colocalizations or associations. These results suggested that SS18 may be directing SS18-SSX fusion proteins to SS18-associated regions in the nucleus where the fusion protein may exert abnormal effects. This was later found to be incorrect, with the *SSX *domain directing the fusion constructs to subcellular locations, as described below. In addition to these findings, dos Santos and colleagues also identified three putative bipartite nuclear localization signals (NLSs) that had not been previously reported. These NLSs, rich in lysine and arginine residues, were distributed along the protein sequence (aa9–23, aa52–64, aa158–172) with NLS3 (aa158–172) completely conserved among the known SSX family members. Following on this work, it was found shortly thereafter that the SS18 domain of SS18-SSX fusion constructs can act as a transcriptional coactivator (70-fold activation) and SSX1 could act as a transcriptional repressor (50-fold repression) when these constructs were coupled to a GAL4 DNA-binding domain in reporter assays [34]. These investigators theorized that the C-terminal SSX domain may actually direct the SS18 activation domain to new or different target promoters. 

Lim and colleagues later confirmed that the SS18-SSX1/2 proteins are transactivators, while the KRAB domains of SSX genes function as low-level transcriptional repressors [35]. In GAL4 repression assays the KRAB domains alone from SSX1 and SSX2 downregulated transactivation of a reporter gene by 3.5- and 3.3-fold, respectively, compared to >83-fold repression for a control KOX1-KRAB domain that has been shown to have potent repression activity [36]. This result further suggested that the SSX-KRAB domain may not function the same way as in other KRAB-containing proteins. Interestingly, the full-length SSX1 protein was able to repress transactivation in the GAL4 assay by 74-fold. They therefore postulated that another repression domain must exist in SSX that is distinct from the KRAB domain. Assaying deletion constructs, they narrowed down the area of the protein responsible for this repression to the last 33 C-terminal amino acids of SSX2. They called this domain the SSX repression domain (SSXRD), which was predicted to fold into an alpha helix. This domain had greater than 95% homology among the first five SSX family members identified at the amino acid sequence level and no homology was found for this domain among any proteins in the ProDom database within SwissProt. Of note, this domain is retained in SS18-SSX fusion proteins and therefore may contribute to the functional activity of this fusion construct. Since the full-length SSX protein was found to have greater repression activity in reporter assays than the SSXRD itself, this suggested that the KRAB domain may augment the activity of the SSXRD. 

The domains of SS18 and SSX responsible for nuclear and spatial targeting were later delineated by dos Santos et al. [37]. Creating a series of FLAG- or VSV-tagged deletion mutants, they showed that the N-terminal amino acids 51 to 90 of SS18 are responsible for its nuclear localization, even though this region does not contain an NLS, whereas SSX nuclear targeting was dependent on the last 34 C-terminal amino acids (SSXRD). Since this domain is conserved between family members in humans and other species, it suggests that this region is used in nuclear targeting for all SSX proteins. The putative NLS3, which was predicted earlier by dos Santos et al. to be the dominant SSX NLS [33], was confirmed by amino acid substitutions to play a role in nuclear targeting. However, knockout of NLS3 resulted in both cytoplasmic and nuclear staining suggesting that SSX may use additional, nonbasic amino acid residues for nuclear targeting. Additionally, it was observed from truncated SS18-SSX constructs that the SSXRD is required for subnuclear localization for the fusion protein, producing the diffuse, even staining and fine punctate dots observed by others [33, 34, 38]. Furthermore, these truncation experiments showed that the SSXRD is responsible for the association of SSX with mitotic chromosomes and localization to sites of Polycomb group silencing complex bodies (PcG bodies) in both transfected cells and 518A melanoma cells, suggesting that these functions of the SSXRD are endogenous. 

 Another SSX domain was also identified by dos Santos et al. which is immediately upstream of the SSXRD and shows relatively high divergence in sequence between SSX family members [37]. They called this sequence the SSX divergent domain (SSXDD), and its function remains unknown.

### 4.2. Interaction with the Polycomb Group Silencing Complex

To further elucidate SSX protein function, Soulez et al. carried out immunolabeling experiments with hemagglutinin (HA)-tagged SS18, SS18-SSX1, SS18-SSX2, SSX1, and SSX2 proteins expressed exogenously in transfected COS-7 cells, the SS cell line CME-1, and in the human fibrosarcoma cell line 2C4 [38]. As observed by dos Santos et al. [33], the expression patterns of SSX1 and SSX2 were diffuse, whereas SS18 constructs were punctate. However, in the Soulez paper, toroidal-like structures were observed in 2C4 cells transfected with SS18 or SS18-SSX1/2, and two prominent foci were observed in the cells transfected with SS18-SSX1/2 or SSX1/2. To determine if these proteins colocalize with any known nuclear compartments, these investigators carried out double immunofluorescence studies with antibodies against SS18, SSX, and other characterized nuclear antigens. Soulez and colleagues confirmed that neither SSX, SS18, nor the fusion constructs localize at sites of active transcription, however, they did find that SS18-SSX1 and SS18-SSX2 expression colocalized with members of the polycomb group (PcG) complex [39]. Specifically, SS18-SSX2 was found to localize to sites of RING1 expression in all cells examined, and SS18-SSX1 and SS18-SSX2 colocalized to sites of endogenous BMI-1 expression (both members of the PcG complex). Further labeling experiments showed no association between SS18 and BMI-1, yet SSX1 and SSX2 were both shown to colocalize with BMI-1. This result suggested that the SSX portion of SS18-SSX directs localization of the fusion constructs within the nucleus to sites of PcG body accumulation, contrary to what was previously theorized by dos Santos et al. and Brett et al. [33, 34]. Specifically, it has been shown that the SSXRD is responsible for association of SSX with PcG proteins and core histones [37, 40]. 

**Figure 2:**
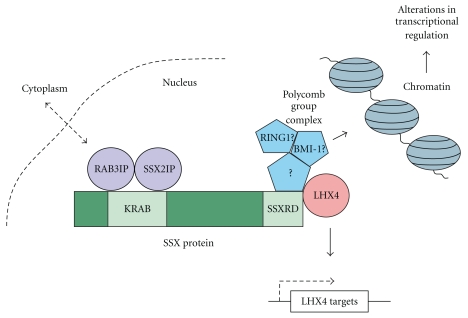
Functional interactions of the SSX proteins. The RAB3IP and SSX2IP protein interaction with the SSX KRAB domain and LHX4 and PcG protein interactions with the SSXRD domain are depicted. Dynamic intracellular and intranuclear interactions are highlighted.

 Although much is still not known about the function of PcG complexes in mammals, it has been demonstrated that these protein complexes have some role in the regulation of cell cycle progression and differentiation [41]. PcG knockout experiments and overexpression studies have shown that PcG genes are associated with proliferation of progenitor cells in the murine haematopoietic system, with BMI-1 overexpression leading to lymphoma development [42, 43]. It has been shown in *Drosophila* that multimeric PcG complexes function to maintain homeotic gene silencing, serving as a stable repressor system to ensure the proper timing of developmental programs [44, 45]. Like SSX proteins, many PcG complex members lack DNA-binding domains and are believed to exert their repressive functions through epigenetic regulation of chromatin structure via protein-protein interactions [46]. Coimmunoprecipitation studies of tagged SSX or SS18-SSX using antibodies for RING-1 and BMI-1, however, showed no *direct* association between these proteins. This result suggested that SSX may interact with other components of the large PcG complexes, perhaps proteins not yet identified (Figure 2). Since it had been previously reported that PcG complexes interact with chromatin throughout mitosis [39], Soulez et al. carried out immunolabeling experiments of transfected cells with propidium iodide-stained chromatin to determine if SSX associates with condensed chromatin [38]. No association was found between SS18 and chromatin distribution, however both SSX1 and SSX2 were found to be distributed evenly over condensed chromosomes during mitosis with especially dense staining during prometaphase and metaphase. Unlike SSX1 or SSX2, SS18-SSX2 associated with prometaphase/metaphase chromosomes in punctate patterns similar to those described for RING-1 and BMI-1 [39], perhaps suggesting a functional difference between wild type SSX and the fusion protein that could potentially lead to SS. 

### 4.3. Interaction with RAB3IP and SSX2IP

Using yeast two-hybrid assays and glutathione-S-transferase (GST) pull-down assays, de Bruijn et al. identified the first two proteins that directly associated with SSX2 [47]. Three clones were identified from the yeast two-hybrid screens, encoding sequences 1.1, 1.5, and 1.7 kb long. The 1.7- and 1.5-kb clones pulled out were sequenced and identified as RAB3IP, a human homologue of the *Rattus norvegicus Rabin3* gene, which shared 84% cDNA and 88% protein identity, respectively. The two different-sized clones were found to be alternative splices of *RAB3IP*, and were designated *RAB3IP* *α* and* RAB3IP* *γ*, with* RAB3IP* *α* encoding a predicted 51 kDa protein 460 amino acids long, which is analogous to Rabin3 in rats. The 1.7- and 1.5-kb inserts also showed homology to the *Pat-12* gene that has been characterized in mice [48]. Both *Rabin3* and *Pat-12* genes are thought to potentially encode Ras-like GTPase-binding proteins, but these proteins are not fully characterized [49]. The 1.1-kb clones identified as SSX2IP were predicted to encode a 71 kDa protein 614 amino acids long, which had no homology to any known sequences. RAB3IP did not interact with SSX1, SSX3, or SSX4, whereas SSX2IP interacted with SSX3 but not SSX1 or SSX4, which may suggest that the SSX members have different functions. By carrying out deletion mutant studies, they also determined that these two proteins interact with the N-terminal moiety of SSX2, with RAB3IP interacting with SSX2 aa 25 to 80 and SSX2IP interacting with SSX2 aa 1 to 80 (Figure 2). Interestingly, the region of SSX2 that interacts with RAB3IP almost exactly overlaps with the SSX2 KRAB domain, which spans aa 20 to 83. Since RAB3IP was found to be expressed in many different tissues by Northern blot analysis and has homology to proteins in species as diverse as *C. elegans* and yeast, it was suggested that RAB3IP may be a house-keeping gene. Murine homologues to SSX2IP were identified from blast analysis and ubiquitous expression was found for this gene as well. The presence of four coiled-coil domains, and its homology to known structural proteins, suggests that SSX2IP may function in nuclear architecture, while the many motifs identified in RAB3IP, including coiled-coiled and ER-retention motifs, indicate that this protein may carry out diverse functions. Interestingly, RAB3IP is found on chromosome 12q13-14, a region known to be frequently rearranged or amplified in human cancers [50, 51], and SSX2IP is on 1p22 also in a region commonly deleted, amplified, or translocated in human tumors [52–54]. Immunofluorescence studies showed that RAB3IP normally localized to the cytoplasm (like Rabin3), but interestingly, when cells were transfected with both RAB3IP and SSX2, 10% of RAB3IP protein translocated to the nucleus, whereas SSX2IP was always shown to colocalize with SSX2 in the nucleus [47].

Further analysis of SSX2IP was recently reported by Breslin and colleagues in a review on SSX2IP and its emerging role in cancer [55]. They report that SSX2IP was identified as a leukemia antigen through a SEREX screen, specifically recognized in the sera of patients with acute myeloid leukemia (AML) as compared to sera from healthy donors, and that SSX2IP expression was found in 33% of leukemic cells from AML patients but not in normal donor hematopoietic cells tested by RT-PCR [56]. SSX2IP has also been shown to be highly expressed on the cell surface of myeloid leukemia cells and AML tissue samples using immunohistochemistry, flow analysis, and confocal microscopy [57]. This surface expression on myeloid leukemia cell lines appeared to peak during mitosis. 

 SSX2IP is also known as human afadin DIL domain-interacting protein (ADIP) for its homology to the mouse and rat ADIP genes, which are 88% and 87% identical to SSX2IP, respectively. Microarray data has demonstrated lower expression of SSX2IP in AML patients with *t*(8;21) translocation, while higher expression of SSX2IP was associated with the *t*(15;17) translocation [58]. Of note, other genes elevated in expression in patients with the *t*(15;17) translocation include proteins involved in cell cycle regulation (e.g., p57^Kip2^, cdk7, cyclins D2, D3, E2 and B2), replication (Cdc6), and mitosis (survivin and CENPJ). In human leukemias, SSX2IP appeared to be expressed in cell cycle-regulated patterns. 

### 4.4. Interaction with LHX4

In an attempt to comprehensively identify proteins that associate with SSX, de Bruijn and colleagues conducted yeast two-hybrid studies and found that one of the clones pulled out encoded the LIM homeobox protein LHX4 [59]. This protein was previously shown to be deregulated or translocated in multiple forms of leukemia [60–64]. The C-terminal SSXRD domains of SSX1, SSX2, and SSX4 all interacted with LHX4 in yeast two-hybrid assays, suggesting that this protein interaction is common to SSX proteins. LHX4 is a 390 amino-acid protein containing two LIM domains (LIM1 and LIM2), a homeobox domain (HOX), two zinc fingers, and was found to have a C-terminal tail with a novel transcriptional activation domain. Utilizing immunofluorescence, de Bruijn and colleagues demonstrated that SSX1 and SS18-SSX colocalized with LHX4 in transfected COS1 and HeLa cells; however, in a small percentage of cells the overlap was not entirely complete suggesting that these interactions between SSX1 and LHX4 are dynamic. The interaction of SSX1 and SS18-SSX with LHX4 was subsequently verified through coimmunoprecipitation analysis. To evaluate whether SSX or SS18-SSX can alter the expression of LHX4 target genes, de Bruijn et al. conducted ChIP and transactivation assays. The glycoprotein hormone-*α* (*CGA*) gene, encoding CGA, is a known target of LHX4 binding and transcriptional regulation [65]. LHX4 was shown to bind directly to the CGA promoter, with increased CGA transactivation in conjunction with SS18-SSX2 in luciferase reporter assays of transfected SYO1 SS cells. Interestingly, in similar assays it was found that SSX2 alone actually had an inhibitory effect on CGA activation, which indicates that SS18-SSX2 and SSX2 have opposite activities of transcriptional regulation in SS. It was proposed in this study that the interaction between SSXRD and LHX4 may be dynamic, with LHX4 competing with PcG proteins for the SSXRD, perhaps altering the transcriptional corepression activities of SSX (Figure 2).

### 4.5. Expression in Mesenchymal Stem Cells

The expression patterns of CTA proteins in testis germline cells that are constantly undergoing proliferation prompted Cronwright and colleagues to evaluate SSX expression in normal somatic cells that are also capable of self-renewal and endless proliferation [66]. Specifically, this group investigated the expression of members of CTA gene families including NY-ESO-1, MAGE-A, GAGE, RAGE, and SSX in human postnatal bone marrow and fetal liver mesenchymal stem cells (MSCs). SSX was found to be expressed in melanoma cells, fetal MSCs, adult MSCs, and bone marrow by RT-PCR. Surprisingly, immunofluorescence analysis of MSCs showed both fine and granulated patterns of SSX expression in the cytoplasm of these cells. Bone marrow smears stained for SSX expression showed a few SSX-positive cells that were also CD34+ (hematopoietic stem cells). Immunostaining the MSCs for CTAs after the cells were allowed to partially differentiate into adipocytes or osteocytes, Cronwright's group observed that the CTA expression was markedly decreased, especially for SSX [66]. This result was confirmed by RT-PCR analysis as well. Decreased levels of SSX expression were also observed in human fetal tissues allowed to differentiate. Double-immunolabeling of SSX with known proteins associated with migration, invasion, and metastasis were also carried out. The specific proteins evaluated were matrix metalloproteinase 2 (MMP2), which cleaves a number of cell matrix proteins and is associated with invasion and metastasis, vimentin, a component of cytoskeleton intermediate filaments, and fibronectin and laminin, involved with cell adhesion. SSX colocalized with both MMP2 and vimentin. SSX expression was observed to overlap with laminin but no direct colocalization was observed, whereas no overlap was observed with fibronectin. Direct associations between SSX and these proteins were not observed from coimmunoprecipitation studies, however it was found that when melanoma cell line DFW (known to express SSX) was knocked out for SSX expression, DFW cells had 40% decreased ability to migrate in soft agar compared to SSX+ DFW cells, and this reduction was accompanied by a decrease in MMP2 expression. Vimentin and E-cadherin levels were not influenced by lack of SSX expression in these cells. Also, it was observed that SSX expression is ablated during MSC differentiation, while MMP2 expression decreases and vimentin levels remain unchanged. Overall, even though SSX was not found to directly interact with MMP2, it does appear to indirectly influence its expression patterns, implicating a link between SSX expression and the processes of self-renewal and tumorigenicity. This study provided the first potential evidence of a link between SSX expression and the processes of cell migration and metastasis. It was also proposed in this study that if CTAs are expressed in stem cells with limitless proliferation potential then their ectopic expression in tumor tissue may be a hallmark of dedifferentiation back into a stem-cell like state, perhaps through an altered regeneration program [66].

## 5. SSX Expression in Testis and Tumor Tissues

The normal expression of SSX mRNA has been shown by multiple investigators to be predominantly restricted to testis germline tissue [21, 22, 27, 67]. SSX protein expression in the testis is predominantly localized to the nucleus of spermatogonia cells and occasionally in spermatocytes as demonstrated by IHC [68]. These expression patterns were heterogeneous, with only a subset of spermatogonia found to have SSX nuclear staining. Constitutive genome-wide demethylation has been demonstrated in spermatogonia cells where SSX proteins are normally expressed [69, 70], and interestingly, these same global demethylation patterns have been observed in tumor tissue and tumor cell lines and are associated with the reactivation of silenced genes [71, 72]. This finding indicates that the same physiological mechanisms that lead to SSX expression in the testis may be ectopically reactivated in tumor tissues. Except for SSX6 and SSX10, expression of all SSX family members has been demonstrated in testis tissue (Table 1) [29, 73]. 

The expression of SSX family members has also been demonstrated ectopically in many types of cancer (Table 2). When Türeci et al. initially identified SSX2 as the tumor antigen HOM-Mel-40, they also reported by RT-PCR and Northern blot that SSX2 mRNA was expressed in the following tumor tissues: melanoma (8/16), colorectal carcinoma (9/35), prostate cancer (5/25), breast cancer (7/36), hepatocellular carcinoma (3/6), glioma (2/23), lymphoma or leukemia (1/9), gastric carcinoma (1/12), and thyroid carcinoma tissue samples (2/4) (Table 2) [22]. In 1996 Türeci et al. carried out a more comprehensive study evaluating SSX family member mRNA expression in cancers of different histological types. In this analysis they found that SSX1, 2, and 4 were expressed in 8%, 15%, and 15% of tumors, respectively, with no detection of SSX3 in any samples and detection of SSX5 in only 7 out of the total 325 tumor specimens evaluated [76]. Specifically, SSX mRNA was expressed in 75% of synovial sarcoma, 57% of head and neck cancer, 55% of bladder cancer, 50% of ovarian cancer, 43% of malignant melanoma, 40% of prostate cancer, 36% of nonHodgkin's lymphoma, 33% of stomach cancer, 27% of colorectal cancer, 21% of breast cancer, 21% of lung, 16% of glioma, 13% of endometrial, and 4% of renal cell carcinoma tissues (Table 2). Expression of more than one SSX family member was found in several tumor types, and altogether 27% of tumor tissues expressed at least one SSX family member. 

In other reports of SSX family member tumor expression, SSX5 expression has been found in multiple myeloma [86], osteosarcoma [89], and hepatocellular carcinoma [81], while SSX1 expression has been found in these tumors as well as nonsmall cell lung cancer, Hodgkin's lymphoma, nonHodgkin's lymphoma, leukemia, bladder cancer, breast cancer, colorectal cancer, endometrial cancer, head and neck cancer, and malignant melanoma [76, 77, 80–84, 86, 88, 89]. SSX2 and SSX4 expression have been found in all of the cancer types listed above as well as neuroblastoma, gastric cancer, ovarian cancer, synovial sarcoma, mesothelioma (SSX2), pancreatic cancer (SSX4), and intrahepatic cholangiocarcinoma (SSX4) (Table 2) [22, 76–92]. SSX expression has also been evaluated by RT-PCR in brain tumors in which SSX1 was only expressed in astrocytomas, while SSX2 was expressed in astrocytomas and oligoastrocytomas, and SSX4 was expressed in both of these as well as oligodendrogliomas [92]. 

Using a novel sequence-based amplification assay to determine SSX mRNA quantities in 211 bone and soft tissue tumor specimens, Naka et al. found that SSX expression levels varied greatly among tumor samples, and they found that malignant tumors showed much higher SSX expression levels than benign tumors (*P* < .0001) [93]. Additionally, stage III tumors had significantly higher SSX mRNA expression levels as compared to stage I or stage II tumors (*P* < .005). These results associated SSX expression with more advanced stage of disease in cancer patients. In another study by Taylor et al. it was found that SSX1, SSX2, SSX4, and SSX5 were all coexpressed in 20% of patients with multiple myeloma (MM), and this coexpression was found to correlate with adverse prognosis and reduced survival (*P* = .0006) [86]. Of these four SSX family members, SSX2 was found to be the most strongly associated with worse prognosis (*P* = .0001). Other MM patients not expressing all four of these SSX family members were often found to express one or more SSX proteins. In addition to MM, SSX2 expression in prostate cancer has been reported by our group to be associated with advanced-stage prostate cancer [74]. 

Like normal testis cells, the overall expression of SSX protein in human tumor tissue samples has been shown to be quite heterogeneous in expression [68]. SSX nuclear staining was found in one report to be present in 24% of melanoma locoregional metastases and 40% of primary melanoma tissue. Within the majority of all lesions less than 25% of the tumor cells were found to be SSX positive. Staining was observed in four main patterns, (a) widespread, with >75% of cells expressing, (b) focal, with clustered positive cells in a limited tumor area, (c) scattered, with a few positive cells localized throughout the lesion, or (d) isolated, with just a few positive cells in several tumor areas. This heterogeneous expression has been a major concern for tumor immunotherapy since the outgrowth of antigen-negative cells might develop with targeted antigen-specific therapy. 

 Since SSX expression has been shown in mesenchymal stem cells [66], melanoma stem cells [94], and only heterogeneously in many tumor types, it may be necessary to use agents to increase SSX antigen expression in tumor tissue to therapeutically target these antigens. Within tumor tissue it may be that cells expressing SSX proteins are stem cells, dividing cells, or those that have malignant potential, and perhaps this is why SSX expression appears so heterogeneous in tumor lesions. These may be precisely the cells of most interest for therapeutic targeting. Conversely, if not all malignant cells express the SSX antigen, then outgrowth of SSX-negative escape variants could potentially develop. Recent work with epigenetic modifying agents may present one possible solution to this problem. In a report by dos Santos et al. it was found that SSX2 mRNA and protein expression could be induced with 5-aza-2′-deoxycytidine (5-aza-dc) treatment in cultured BLM melanoma cells (SSX-negative) and K562 erythroleukemia cells (SSX-positive) [68]. Specifically, RT-PCR showed that SSX mRNA expression was clearly induced in BLM cells and increased in K562 cells with 5-aza-dc treatment, while SSX protein expression was found by immunofluorescence staining in 9–13% of all treated cells. No protein expression was found in the absence of 5-aza-dc treatment. SSX expression has also been shown to be upregulated with 5-aza-dc in bladder cancer cell lines that are SSX-negative prior to treatment [95], and SSX2 has been shown to be inducible with 5-aza-dc in mesothelioma [85]. We have also reported that SSX2 expression can be upregulated selectively in prostate cancer cell lines, but not normal prostate epithelial cells, by treatment with 5-aza-dc [74]. In a report by Güre et al. it was found that SSX4 was inducible with the histone deacetylase inhibitor Trichostatin A (TSA) in melanoma cell lines, with minor effects for SSX2 and SSX6, whereas 5-aza-dc was able to more effectively induce expression of SSX1, 2, 4, and 5 in melanoma cell lines [29]. SSX6 expression was only slightly inducible with 5 aza-dc in these studies, and overall, SSX4 was the most frequently induced family member in these particular cells, followed by SSX2, SSX5, SSX1, and SSX6 (Table 1). Finally, in another report by Sigalotti et al. it was shown that upregulation of SSX expression in melanoma cell clones with 5-aza-dc treatment was directly correlated with promoter demethylation patterns. This finding provided a clear mechanism of action for upregulating CTA expression in cancer cells with DNA methyltransferase inhibitors, which was thought to explain the intratumoral heterogeneity of CTA expression patterns, and provided further support for the use of pharmacologic agents to upregulate antigen expression on tumor cells for targeted tumor immunotherapy [96]. 

## 6. SSX Immunology

The interest in SSX proteins as immunotherapeutic targets began with the identification of SSX2 as the tumor antigen HOM-Mel-40 [22], which had been shown to be the target of humoral immune responses in as many as 10% of patients with melanoma [22, 97, 98]. In other studies humoral SSX2 immune responses were observed in 2 out of 74 colon cancer patient sera samples [99], 1 out of 100 prostate cancer patient sera [74], and antibody responses to SSX4, but not SSX2, have been identified in 2 out of 131 patients with gynecological cancers but not in healthy controls [100]. SSX common antigen antibody responses have also been detected in sera from pancreatic, lung, breast, colon, and ovarian cancer patients but not in healthy control individuals [101]. Additionally, antibodies specific for SSX1, 2, 3, and 4 have been found in the sera of cancer patients with melanoma, colon cancer, and/or breast cancer [27, 99, 102]. In another study by Valmori et al. analysis of SSX4 IgG immune responses in epithelial ovarian cancer (EOC) patients showed that two patients out of 109 had both SSX2 and SSX4 IgG antibody responses [103]. These responses were confirmed by ELISA, and both patients with dual antibody responses were alive and showed antibody responses >5 years after initial therapy.

Observing that cancer patients can have preexisting SSX2 IgG-specific immune responses, other investigators sought to identify if cancer patients can have cell-mediated immune responses to SSX proteins. Ayyoub et al. carried out the first investigation to identify SSX2 T-cell epitopes by utilizing an altered reverse immunology strategy [104]. Purified standard proteasome complexes from human erythrocytes were incubated *in vitro* with a library of overlapping SSX2 peptides covering the entire 188 aa protein sequence. The digested products were analyzed by mass spectrometry, which identified 12 peptides that were processed from the proteasome: SSX2p5–13, p7–15, p15–24, p16–24, p40–49, p41–49, p50–59, p53–61, p57–65, p58–67, p59–67, and p103–111. These peptides were subsequently cultured with cells from the tumor-infiltrating lymph node (TILN) of an SSX2-positive metastatic melanoma lesion (LAU 50), and an IFN*γ*-ELISPOT was performed to detect SSX2 peptide-specific T-cell immune responses. SSX2 peptide p41–49 elicited the greatest T-cell immune response from the TILN, which led the group to synthesize HLA-A2 multimers containing SSX2 peptide p41–49 (Table 3). These multimers were used to stain the TILN cells and showed clearly positive SSX2 p41–49-specific CD8+ T cells. It was also shown that SSX2p41–49 CD8+ T cells (CTL clone LAU 50 E2.4) could lyse peptide-pulsed T2 cells and HLA-A2+ melanoma cell lines. Interestingly, when this clone was incubated with COS7 cells transfected with plasmids encoding HLA-A2 and SSX2 or SSX4, high levels of IFN*γ* secretion and lysis were observed by the clone with SSX2-transfected cells but not SSX4-transfected cells. Since SSX2 and SSX4 only differ by 2 aa within the antigenic epitope region, this result suggested high specificity of the clone for SSX2 p41–49. None of the other predicted SSX2 epitopes were recognized by TILN from this patient.

Following on this work, Ayyoub et al. utilized fluorescent HLA-A2/SSX2p41–49 multimers to determine the relative frequencies of SSX2 p41–49 CD8+ T cells in melanoma patients with SSX2-positive or negative tumors and in healthy donors [105]. They found that SSX2 p41–49-specific CTLs were identifiable in both melanoma patients and healthy donors, although at lower frequency in healthy donors or patients with SSX2-negative tumors. They showed that p41–49-specific CTLs from the TILN and peripheral blood mononuclear cells (PBMCs) of patients with SSX2-expressing tumors were better able to lyse tumor cells compared to CTLs from the TILN and PBMCs from patients with SSX2-negative tumors or PBMCs from healthy donors. It was also shown that SSX2-specific CTL frequency increased with disease progression in at least one melanoma patient. SSX2 p41–49 T cells have also been identified in patients with hepatocellular carcinoma (HCC) [116]. These CD8+ T cells were identified using SSX2 p41–49/HLA-A2 multimers in one out of six patients with HCC, both in the TILN and PBMCs. A polyclonal SSX2 p41–49 T-cell line generated from this patient lysed p41–49-pulsed T2 cells, melanoma cell line MEL 275, and a p41–49 peptide-pulsed HCC cell line. This was the second solid human malignancy with evidence of recognition by SSX2 p41–49-specific CTL.

The immunogenicity of SSX2 p41–49 was later highlighted when Rubio-Godoy and colleagues identified this peptide through an approach called positional scanning of synthetic combinatorial peptide library analysis [117]. In this report, 3.1 × 10^11^ nonamer peptides, arranged in a positional scanning format, were screened for melanoma-reactive CTL of unknown specificity and assessed for their ability to elicit peptide-specific CTL. The identified optimal peptide sequence (AAAPKIFYA) was very similar to SSX2 peptide p41–49 (KASEKIFYV). Recognition of this epitope by melanoma-reactive CTL clone LAU 50/4D7 was confirmed by cytotoxicity assay, and interestingly, the native SSX2 peptide p41–49 had recognition that was in the same range as the optimal peptide. Attempts to modify the anchor residues of the optimally identified peptide or the native p41–49 epitope did not result in enhanced peptide recognition. Additionally, no cross-reactivity of p41 from SSX1, SSX3, SSX4, or SSX5 was observed. Further, a biometrical analysis of the screening data was used to generate a scoring matrix of all predicted peptides in public protein databases that could potentially be recognized by clone LAU 50/4D7, and, amazingly, this approach ranked SSX2 p41–49 27th out of approximately 16 million nonamer peptides [118]. If this analysis was restricted to known tumor antigens in humans, this rank was 2nd out of 400,271 peptides. Ranked scores for other SSX peptides were much lower, however. It was noted that this method of scoring does not omit other SSX peptides as potentially immunogenic, but it does emphasize the immunogenicity of SSX2 p41–49. 

In an effort to identify additional class I MHC-restricted SSX2 epitopes, Wagner et al. used a reverse immunology approach to identify SSX2 epitopes that were predicted to have affinity for the human HLA-A2 MHC class I complex using the SYFPEITHI algorithm [108, 119]. PBMCs from seven breast cancer patients and eleven healthy donors were evaluated for reactivity to peptides p41–49 and p103–111, and p167–175, which had all been predicted from the peptide-binding algorithm to have affinity for HLA-A2. They found that 5/7 (71%) of the HLA-A2+ breast cancer patients and 6/11 (55%) of the HLAA2+ healthy controls had T cells that were reactive to SSX2 peptide p103–111 by IFN*γ*-ELISPOT assay. HLA-A2 restriction of the responses was verified using specific HLA blocking antibodies. SSX2 p103–111 was also shown to be a naturally presented SSX2 epitope by the recognition of SSX2+ SK-MEL-37 melanoma cell line and transfected COS7 cells by p103–11 peptide-specific CD8+ T cells (Table 3). Also, SSX2 p103–111-specific T cells could lyse peptide-pulsed target cells in cytotoxicity assays. Interestingly, no association was found between SSX2 antibody titer in breast cancer patient serum and p103–111-specific T-cell responses, indicating that the humoral and cell-mediated responses to this antigen are regulated independently. Rentzsch and colleagues found that PBMCs from one out of ten primary breast cancer patients exposed to SSX2-p103–111 significantly increased their mRNA expression of IFN*γ* by QT-RTPCR analysis [109]. This immune response was also detected in one of eleven healthy control individuals. We have also recently identified SSX2 p103–111 as an SSX2 epitope using HLA-A2 transgenic mice immunized with a genetic vaccine encoding SSX2, and further identified that p103–111-specific CTL can lyse HLA-A2-expressing prostate tumor cells (Smith, manuscript submitted).

Not only has the endogenous processing and presentation of SSX2 peptide p103–111 been demonstrated by reactivity of p103–111-specific CTL for SSX2-expressing tumor cells, it has also recently been shown that this peptide epitope is directly presented on the surface of cancer cells [73]. By utilizing a human phage library screening technique Held et al. generated SSX2 p103–111-specific Fab antibodies that specifically recognized and bound to this peptide in the context of HLA-A2. These antibodies were used to stain melanoma cell lines in fluorescence microscopy studies, and it was observed that a majority of SK-Mel-37 cells expressed p103–111/HLA-A2, whereas Me275 cells expressed very little (<1% of cells). These cells were also used to stimulate SSX1 p103–111-specific T cell clones, and it was determined that the expression of p103–111/HLA-A2 on the cell surface, and not the total SSX2 protein expression levels of the cells, was correlated with enhanced CTL recognition and activation. 

Ayyoub et al. also identified the first CD4 class II MHC SSX2 epitope [110]. This epitope was mapped to aa 19–34 of SSX2 using truncated peptide assays and was recognized by CD4+ T cells from an SSX2-expressing melanoma patient (Table 3). No responses to this epitope were found in healthy controls, and class II blocking antibody experiments verified that this epitope is recognized in the context of HLA-DPB1*0101. Many studies have documented the atypical expression of class II MHC molecules on the surface of colon carcinomas, breast cancer cells, sarcomas, and melanoma cells [120–124]. This expression prompted the investigators in the current study to evaluate whether SSX2 p19–34 is processed endogenously and presented on the surface of tumor cells. Interestingly, SSX2 CD4+ T cells failed to recognize IFN*γ*-treated melanoma cells expressing SSX2 and class II MHC molecules (HLA-DP); however, they were stimulated by antigen-loaded dendritic cells. This result suggests that p19–34 may not be endogenously presented on tumor cells but *can* be loaded exogenously and presented by APCs. Ayyoub et al. also identified an HLA-DRB*1101-restricted SSX2 class II peptide p45–59 [112]. CD4+ T cells specific for this epitope were detected among PBMCs and in the TILN from melanoma patients but not in healthy control individuals. Using the SYFPEITHI algorithm, p45–59 was ranked as the 2nd highest predicted HLA-DR-binding peptide for SSX2, whereas this peptide ranked 3rd, 13th, and 2nd for SSX1, SSX4, and SSX5, respectively. Titrated concentrations of peptides incubated with p45–59-specific CD4+ T cells showed cross-reactivity to SSX5 p45–59 and low-level reactivity to SSX1 p45–59 in IFN*γ* ELISA assays. Eleven of nineteen melanoma patients had CD4+ T cells responses to this epitope region (i.e., aa 37–58). Contrary to what was found for the class II peptide p19–34, T cells recognized epitope p45–59 on both antigen-loaded DCs as well as HLA-DR+ tumor cells. This peptide was also described by Neumann and colleagues in which p45–59 was found to be restricted to HLA-DRB1 subtypes *0701, *1101, *1302 and B3*0301, and it was demonstrated that p45–59 CD4+ T cell responses could be induced in 3/6 of breast cancer patients and 1/5 of healthy controls [113]. However, no correlation was found between SSX2 antibody titer and CD4+ T cell responses.

After the identification of the first two class II SSX2 epitopes Ayyoub and colleagues identified yet another CD4 peptide that overlapped with these epitopes [111]. This epitope region was narrowed down to amino acids p37–51 of SSX2 by truncation experiments and was shown to be restricted to HLA-DRB*0301 by class II MHC blocking antibody assays. Since this was the third immunogenic peptide identified in the KRAB domain region of SSX2, it was theorized that this region may be a “hot spot” for T-cell recognition. Again, wild type melanoma cell lines expressing MHC class II molecules or tumor cells transfected with plasmid encoding SSX2 were not recognized by epitope-specific CD4+ T cells directly, however, p37–51 peptide-pulsed tumor cells *were* recognized by both SSX2 p37–51-specific CD4+ T cells and SSX2 p41–49 peptide-specific CD8+ T cells. Of note, the transfected cells were recognized by the p41–49-specific T cells but not the p37–51-specific CD4+ T cells, confirming that the p41–49 peptide can be endogenously processed and presented by tumor cells. Cross reactivity of SSX2 p37–51-specific CD4+ T cells was observed for SSX4 and SSX5 but not SSX1 or SSX3 p37–51 peptides in an IFN*γ* peptide titration ELISA assay.

Since it was previously observed that SSX4 was expressed in ~20% of malignant melanomas in a report by Türeci et al. [76], Ayyoub and colleagues also evaluated the presence of CD4+ T cells specific to this antigen in melanoma patients by stimulating CD4+ T cells isolated from the PBMCs of four melanoma patients with overlapping peptides derived from the amino acid sequence of SSX4 in the presence of APCs [115]. Intracellular IFN*γ* cytokine staining revealed that all four patients had CD4+ T cells that were activated in the presence of at least one of the SSX4 peptides (Table 3). Again, as was found for the SSX2 class II peptides, CD4+ T cells could not recognize melanoma cells directly but were activated by APCs loaded with SSX4 antigen, which suggested that this peptide may not be presented through the endogenous pathway in tumor cells. Interestingly, five out of the seven peptides identified in this study mapped to the KRAB domain of SSX4. Four previously identified SSX2 epitopes also mapped to this region, further suggesting that this may be a “hot spot” for T-cell recognition. Valmori et al. found that epithelial ovarian cancer (EOC) patients also had SSX4 CD4+ T cells that recognized some of these epitopes [103]. 

Several of the SSX4 class II peptides that were identified previously were later reported by Godefroy et al. to also be epitopes encoded from the SSX1 protein [114]. In this study an overlapping pool of peptides spanning the entire SSX1 protein sequence was incubated with PBMCs from cancer-free individuals; SSX1-peptide-specific CD4+ T cells were identified in 5/5 of the donors analyzed. These T-cell populations almost exclusively recognized epitopes from three defined regions of the protein sequence, including the KRAB domain and two C-terminal regions of the protein that are retained in the SS18-SSX fusion construct (Table 3) [115]. Evaluating the cross-reactivity of these peptide-specific clones for other SSX family members, however, revealed only limited recognition of p41–60 for SSX5. This lack of cross-reactivity was explained by the presence of several amino acid differences in this epitope region between family members. As before, no endogenous processing and presentation of SSX1 epitopes by tumor cells was demonstrated. 

 Since it had been shown that tumors can express more than one SSX family member at the same time and that T-cell responses can be found to multiple SSX family members in cancer patients, He et al. assessed the utility of an altered peptide ligand strategy to identify class I epitopes that could be used to target multiple SSX family members [107]. Using peptide prediction algorithms He and colleagues identified SSX p57–65 and p99–107 as shared epitopes between family members SSX1–9. Since SSX p57–65 had the higher binding score for all nine family members, this peptide was selected for further analysis. Using T2 binding assays with four altered p57–65 peptides specific to SSX1–9, it was shown that altered peptide P4 (AMTKLGFNV), which is encoded by SSX6 and SSX8, had the greatest *in vitro* binding affinity for HLA-A2, and this affinity was stable at low peptide concentrations. All four altered p57–65 peptides were shown to elicit peptide-specific CTL from the PBMCs of healthy HLA-A*0201 individuals, however P4-specific CTL showed the greatest lysis and IFN*γ* secretion when incubated with peptide-pulsed target cells. Additionally, P4 showed the greatest cross-reactivity to other altered p57–65 peptides presented by target cells in cytotoxicity assays. HLA-A2.1/K^b^ mice immunized with P4 generated CTL that could be cultured *ex vivo*, and were shown to also lyse T2 cells displaying the immunizing peptide or the three other altered p57–65 peptides, which suggests that this altered peptide strategy might be successfully utilized to target multiple SSX family members (Table 3). 

## 7. Concluding Remarks and Future Directions

The SSX proteins are a highly homologous group of CT-X antigens with demonstrated immunogenicity in patients with cancer, and a number of characteristics that make them attractive targets for tumor therapy. Cheever et al. recently reported that SSX2 in particular may be a high priority target for cancer therapy based upon certain predefined criteria for prioritization of tumor antigens, such as tumor specificity, oncogenicity, expression level, and number of identified epitopes [14]. As essentially tumor-specific antigens from the immunological perspective, SSX proteins may therefore represent ideal targets for tumor immunotherapy. 

As described previously, several groups have demonstrated that SSX mRNA or protein expression in tumors is correlated with more advanced stages of disease and a worse patient prognosis. More work needs to be done to evaluate the function of SSX proteins in cancer to determine if the expression of these proteins is a bystander effect of epigenetic reprogramming or whether the proteins themselves have inherent transforming activity and contribute to the cancer phenotype. For instance, it has been shown that SSX proteins in mesenchymal stem cells colocalize with MMP2, and in knock-down studies it appears that SSX expression is correlated with an invasive phenotype [66]. Additionally, the presence of SSX in the SS18-SSX fusion construct that is expressed in >95% of SS appears to contribute to transformation as well [15], and may be a reasonable target for drug or vaccine-based therapy. Although this fusion protein is expected to have a different function than native SSX proteins, the frequent association of SSX proteins with a cancer phenotype suggests a possible functional role leading to tumor formation. 

Due to their high degree of homology and the identification of conserved “hot spots” of epitope recognition, it might be feasible to target multiple SSX family member proteins simultaneously in tumors coexpressing these SSX family members. For instance, it may be possible to elicit immune responses to these proteins by immunizing patients with vaccines encoding native SSX peptides or protein, or modified vaccines designed to target several SSX family members. Moreover, the ability to modulate SSX protein expression with epigenetic modifying agents might provide a means to increase SSX expression in tumor tissue for targeted immunotherapeutic approaches. However, until it has been established whether SSX proteins contribute to oncogenicity, it may not be prudent to use epigenetic means to increase the expression of these proteins in human patients for clinical immunotherapy trials before it can be shown that this expression would not be harmful. In regard to concerns of transforming activity by the SSX proteins, the ideal immunization platform for therapeutic vaccines may be either SSX peptides or a genetic vaccine encoding a rearranged version of SSX cDNA. This could potentially allow for the transcription and translation of key SSX epitopes that can be presented to T cells without the potential harmful effects that might develop from direct vaccination with SSX proteins or with gene-based vaccines encoding the native protein. 

 The identification of several orthologues of SSX proteins in a variety of mammalian species may provide additional model systems to evaluate the efficacy of targeting these antigens therapeutically. Since these orthologues appear to have similar expression patterns in testis and tumor tissue, it is likely that SSX proteins have conserved evolutionary function, and further investigation of these proteins in other species may shed light on their mechanistic roles in both normal and malignant tissues. Based upon the available information gathered for these proteins, either drug-based therapies or vaccine-based immunotherapies could potentially be developed to treat patients with tumors expressing these antigens.

## Figures and Tables

**Table 1:** SSX family members: expression, fusion constructs, inducibility, and isoforms. The mRNA expression patterns of the ten known SSX family members are shown for testis and tumor tissues in columns 2, 3, and 4, while SSX family members known to be involved in the SS18-SSX fusion event are shown in column 5. SSX members shown to be inducible with epigenetic modifying agents or known to have alternative splice isoforms are shown in columns 6 and 7, respectively. References are given in column 8.

	Transcribed	Expressed in testis	Expressed in cancer	Fusion partner with SS18	Inducible	Alternative splices	Reference
SSX1	+	+	+	+	+		[27]
SSX2	+	+	+	+	+	+	[27, 74]
SSX3	+	+	−*		−		[27]
SSX4	+	+	+	+**	+	+	[27]
SSX5	+	+	+/−		+	+	[27]
SSX6	+	−	−*		+/−		[29]
SSX7	+	+	−*		−	+	[29]
SSX8	+	+	−		−		[29, 73]
SSX9	+	+	−		−		[29, 73]
SSX10	+	n.d	n.d	n.d.	n.d.	n.d.	—

+ = positive/strong, +/− = positive/weak, − = negative/undetectable.

*very seldom.

**Only one case observed [75].

n.d. = not demonstrated.

**Table 2:** Expression of SSX family members in cancers of different histological types. The expression of SSX family members 1–10 are shown for 24 cancers of varying histological origin and in normal testis tissue. “+” indicates that the presence of SSX mRNA or protein has been detected in these tumor tissues.

	SSX1	SSX2	SSX3	SSX4	SSX5	SSX6	SSX7	SSX8	SSX9	SSX10	References
Bladder cancer	+	+		+							[76]
Breast cancer	+	+		+							[22, 76–78]
Cholangiocarcinoma				+							[79]
Colorectal carcinoma	+	+		+	+						[22, 76, 80]
Endometrial cancer	+	+		+	+						[76]
Gastric carcinoma		+		+							[22, 76, 78]
Glioma		+		+							[22, 76]
Head and neck cancer	+	+		+	+						[76]
Hepatocellular carcinoma	+	+		+	+						[22, 81]
Hodgkin's lymphoma	+	+		+							[82]
Leiomyosarcoma											[76]
Lymphoma/Leukemia	+	+		+							[22, 76, 83]
Lung cancer	+	+		+	+						[22, 76, 84, 85]
Melanoma	+	+		+	+	+*	+*				[22, 29, 76]
Multiple myeloma	+				+						[86]
Neuroblastoma		+		+							[87]
Non-Hodgkin's lymphoma	+	+		+							[88]
Osteosarcoma	+	+		+	+						[89]
Ovarian cancer				+							[22, 76]
Pancreatic cancer				+							[90, 91]
Prostate cancer		+									[22, 74, 76]
Renal cell carcinoma		+									[22, 76]
Synovial sarcoma		+		+	+						[76]
Thyroid cancer		+									[22, 76]
Normal testis tissue	+	+	+	+	+		+	+	+		[29, 73]

*Only observed in cell lines.

**Table 3:** SSX epitopes: sequences, haplotype restrictions, and recognition. The known SSX immunogenic class I and class II MHC peptides or epitopes are shown. Amino acid sequence, haplotype restriction, tumor presentation, family member recognition, and references are outlined for each peptide/epitope. Symbol designations include + = SSX4 sequence, n.d. = not demonstrated. ^†^ = p41–49 epitope shown to be recognized on Me 275, SK-MEL-37, T343B, and T567A melanoma cells and SW 872 liposarcoma cells; p103–111 shown to be recognized on SK-MEL-37 melanoma cells and LNCaP prostate cancer cells; and p45–59 shown to be recognized on Me 275 cells. * = Recognized on antigen-loaded dendritic cells but not endogenously presented by tumor cells.

Class	Name	Native peptide sequence	Haplotype	Naturally presented by SSX+ tumor cells	Known family member recognition	Reference
I	p41–49	KASEKIFYV	HLA-A*0201	Y^†^	SSX2	[104–106]
	p57–65	AMTKLGFKA	HLA-A*0201	n.d.	SSX1–9	[107]
	p103–111	RLQGISPKI	HLA-A*0201	Y^†^	SSX2	[108, 109]

II	p19–34	EKIQKAFDDIAKYFS	HLA-DPB1*0101	n.d.*	SSX2	[110]
	p37–51	WEKMKASEKIFYVYM	HLA-DR3*0301	n.d.*	SSX2, SSX4, SSX5	[111]
	p45–59	KIFYVYMKRKYEAMT	HLA-DRB1*1101, *0701, *1101, *1302, and B3*0301	Y^†^	SSX2	[112, 113]
	p21–40	RSKAFDDIATYFSKKEWKKM	HLA-DRB1*1501	n.d.*	SSX1	[114]
	p31–50	YFSKKEWEKMKSSEKIVYVY+	HLA-DRB*0301 and *1101 (SSX4)	n.d.*	SSX1, SSX4	[114, 115]
	p41–60	KSSEKIVYVYMKLNYEVMTK+	HLA-DRB1*1501 or DRB5*0101 (SSX4) and HLA-DR1*1601 (SSX1)	n.d.*	SSX1, SSX4	[114, 115]
	p51–70	MKLNYEVMTKLGFKVTLPPFM+	HLA-DRB1*0701 (SSX4)	n.d.*	SSX1, SSX4	[103, 114, 115]
	p61–80	LGFKVTLPPFMRSKRAADFH	HLA-DRB1*1101	n.d.*	SSX4	[115]
	p101–120	FGSLQRIFPKIMPKKPAEEE+	HLA-DRB1*1101 (SSX4)	n.d.*	SSX1, SSX4	[114, 115]
	p141–160	PPGKANISEKINKRSGPKRG	HLA-DR1*1601	n.d.*	SSX1	[114]
	p151–170	INKTSGPKRGKHAWTHRLRE	HLA-DPB1*1001	n.d.*	SSX4	[115]
	p161–180	KHAWTHRLRERKQLVVYEEI	HLA-DRB1*08 and HLA-DRB3*0202	n.d.	SSX4	[103]

## Abbreviations

SSX: Synovial sarcoma X breakpoint
CTA: Cancer testis antigen
SS: Synovial sarcoma
CTL: Cytotoxic T lymphocyte
PBMC: Peripheral blood mononuclear cell
TAA: Tumor associated antigen
MHC: Major histocompatibility complex
5-aza-dc: 5-Aza-2′-deoxycytidine
HLA: Human leukocyte antigen.
